# Novel Threshold Changeable Secret Sharing Schemes Based on Polynomial Interpolation

**DOI:** 10.1371/journal.pone.0165512

**Published:** 2016-10-28

**Authors:** Lifeng Yuan, Mingchu Li, Cheng Guo, Kim-Kwang Raymond Choo, Yizhi Ren

**Affiliations:** 1 School of Software Technology, Dalian University of Technology, Dalian, 116620, China; 2 Key Laboratory for Ubiquitous Network and Service Software of Liaoning Province, Dalian, 116620, China; 3 School of Cyberspace, Hangzhou Dianzi University, Hangzhou, 310018, China; 4 Department of Information Systems and Cyber Security, University of Texas at San Antonio, San Antonio, TX 78249-0631, United States of America; 5 School of Information Technology and Mathematical Sciences, University of South Australia, Adelaide, 5095, Australia; West Virginia University, UNITED STATES

## Abstract

After any distribution of secret sharing shadows in a threshold changeable secret sharing scheme, the threshold may need to be adjusted to deal with changes in the security policy and adversary structure. For example, when employees leave the organization, it is not realistic to expect departing employees to ensure the security of their secret shadows. Therefore, in 2012, Zhang et al. proposed (*t* → *t*′, *n*) and ({*t*_1_, *t*_2_,⋯, *t*_*N*_}, *n*) threshold changeable secret sharing schemes. However, their schemes suffer from a number of limitations such as strict limit on the threshold values, large storage space requirement for secret shadows, and significant computation for constructing and recovering polynomials. To address these limitations, we propose two improved dealer-free threshold changeable secret sharing schemes. In our schemes, we construct polynomials to update secret shadows, and use two-variable one-way function to resist collusion attacks and secure the information stored by the combiner. We then demonstrate our schemes can adjust the threshold safely.

## Introduction

Rapid advances in Internet technologies have resulted in significant changes in our society (e.g. digitalization of our society), but there are also associated security and privacy risks. In an open communication network, for example, data can be easily intercepted, modified, and even deleted by one or more attackers. It is, therefore, of little surprise that cyber security is a topic of current interest in different disciplines [1, 2]. For example, Javanmardi et al. [3] proposed a fuzzy reputation-based model for trust management in semantic P2P grids, and Li et al. [4] proposed a trust management scheme designed to resist malicious attacks and evaluate the trustworthiness of both data and mobile nodes in securing vehicular ad hoc networks. Butun et al. [5] proposed a cloud-centric, multi-level authentication as a service approach to address both scalability and time constraints for secure public safety device networks. Other research efforts include those reported in [6–9].

Cryptography is an important tool used to ensure data security (e.g. confidentiality). However, the security level is generally determined by the security level of the stored secret key. In 1979, Shamir [10] and Blakley [11] independently proposed (*t*, *n*) threshold secret sharing (TSS) scheme designed to protect the secret by distributing a secret among a group of *n* participants. Only *t* or more participants in this group can cooperate to recover the secret. The (*t*, *n*) threshold secret sharing scheme has been used in various applications, such as in banks to protect the master key, and in certification authorities to protect the private root certificate keys. We refer interested reader to [12–14] for surveys of (*t*, *n*) threshold secret sharing schemes.

In practice, the threshold may have to be adjusted if there are changes in the security policies and adversary structures prior to recovering the secret. Examples of changes that require threshold to be adjusted include: (1) an increase or decrease in the importance level of the secret; (2) a change in participants number (i.e., one or more participants joining or leaving the group); (3) a change in the level of mutual trust between participants; and (4) the leakage of some participants’ secret shadows. In 1989, Laih et al. [15] proposed the first threshold changeable secret sharing (TCSS) scheme to solve this problem. Since then, several other TCSS schemes based on different methods, such as polynomial interpolation [16–18], lattice basis reduction [19–21], and random noise [22], have been proposed in the literature.

In a naive implementation of the TCSS scheme, a dealer constructs new secret shadows for participants in the new access structure once the threshold is changed. Thus, the dealer needs to hold the secret online and the attacker only needs to defeat the dealer to obtain the secret. To avoid such an attack, Desmedt and Jajodia [23] used the secret shadow redistributing technique in their proposed TCSS scheme, which does not require the dealer’s participation after the initialization phase. Similar schemes have also been presented in [24, 25]. In these schemes, each original secret shadow needs to be split into smaller shadows, which are redistributed to all participants in the new access structure. Each participant *P*_*i*_ combines all received smaller secret shadows into one new secret shadow *s*_*i*_′ using a suitable linear combination; thus, each participant only needs to store *s*_*i*_′. Note that all participants are required to simultaneously maintain mutual secure communication channels. However, this may be impractical when the threshold changes, especially when the change is sudden.

To avoid the requirement of maintaining mutual secure communication channels, several TCSS schemes [16, 17, 26–28] based on broadcasting were proposed. In the schemes described in [17, 26], the dealer validates the new threshold by broadcasting a suitable number of her/his own redundant secret shadows. For example, in the scheme of [26], the dealer constructs a (*n* + 1, 2*n*) threshold scheme with *n* redundant secret shadows, and then, sends *n* normal secret shadows to *n* participants. If the threshold needs to be changed to *t*′, the dealer broadcasts *n* − *t*′ + 1 redundant secret shadows. Then, *t*′ or more participants can reconstruct the secret by providing their own secret shadows. In other schemes, in order to validate the new threshold, the dealer broadcasts special information, such as a mask code for the secret [27] and a key for encrypting/decrypting secret shadows [16]. In these schemes discussed, the dealer prepares all secret shadows (also known as advance secret shadows) for potential changeable thresholds during the initialization phase.

Other efforts have also been made on the security and application of TCSS techniques. In 2013, for example, Rao et al. [29] proposed a dynamic threshold multi-secret sharing scheme using Pell’s Equation with Jacobi symbol. In their scheme, participants can verify their secret shadows, which avoid the situation of participants receiving the nugatory information given by the dealer. More recently, in 2015, Wang et al. [30] proposed a dynamic threshold changeable multi-policy secret sharing scheme, based on RSA cryptography and discrete logarithm technique. Their scheme reduces the communication costs and can resist multiform cheating. In the same year, Harn and Hsu [31] proposed a threshold changeable secret sharing scheme based on bivariate polynomial, designed to protect the reconstructed secret from illegal participants.

Zhang et al. [16] proposed (*t* → *t*′, *n*) and ({*t*_1_, *t*_2_,⋯, *t*_*N*_}, *n*) TCSS schemes (hereafter referred to as TCSS-A and TCSS-B schemes). The TCSS-B scheme was the first scheme that could resist collusion attacks launched by participants who have historical secret shadows. However, their schemes suffer from a number of limitations, namely: strict limit on threshold values, large storage space requirement for secret shadows, and significant computation requirement for constructing and recovering polynomials. Thus, in this paper, we propose two improved dealer-free threshold changeable secret sharing schemes, DTCSS-A and DTCSS-B schemes. In our schemes, we construct polynomials to update secret shadows, and use two-variable one-way function to resist collusion attacks and protect the information stored by the combiner. Compared with Zhang et al.’s schemes, our schemes have following advantages:

1. No limitation on threshold values. New threshold *t*′ must be greater than initial threshold *t* in the TCSS-A scheme, and *N* potential thresholds *t*_1_, *t*_2_, ⋯, *t*_*N*_ must satisfy 0 < *t*_*i*+1_ − *t*_*i*_ < *t*_1_ (*i* = 1, 2, ⋯, *N*−1) in the TCSS-B scheme. However, such limitations are avoided in our schemes.
2. Only one shadow storage requirement. Each participant needs to store *t*′ − *t* + 1 secret shadows in the TCSS-A scheme and *N* secret shadows in the TCSS-B scheme. In our schemes, only one secret shadow needs to be stored.
3. Less computation. A total of *t*′ − *t* + 1 polynomials need to be constructed and recovered in the TCSS-A scheme, and *N* polynomials need to be constructed and recovered in the TCSS-B scheme. In our schemes, only one polynomial needs to be constructed and recovered. Thus, our schemes require significantly less computational effort.
4. Dealer-free. Zhang et al.’s schemes require the dealer’s assistance in the running phase, unlike our schemes. Thus, our schemes can reduce the single point-of-attack risk (i.e., attackers only need to target the dealer in the attempt to obtain the secret).
5. Secret shadow reusability. In our scheme, the secret shadow can be reused in new secret reconstruction; thus, increasing the efficiency.

The rest of this paper is organized as follows. Section 2 introduces related concepts, two-variable one-way function and the obligations of participants. The proposed threshold changeable schemes are presented in Section 3. In Section 4, we demonstrate the security of our schemes, and evaluate the performance of our schemes with those of Zhang et al.’s. We also discuss how our schemes can deal with the situation where the threshold needs to be adjusted. Section 5 concludes the paper.

## Preliminaries

In this section, we explain the relevant concepts, two-variable one-way function and the obligations of participants.

### Conceptions

We introduce the related conceptions as follows:

(1) Communication modes

Two communication modes are used in TCSS schemes (i.e. secure communication channels and broadcasting). It may be impractical to maintain mutual secure communication when the threshold changes, especially when the change is sudden. In our schemes, important information such as real secret shadows are sent using RSA-based technique, and we validate the new threshold using broadcasting. We refer interested readers to [32–34] for an overview of secure communication techniques, as this is beyond the scope of this paper.

(2) Dealer-free

TSS technology is generally used to protect the secret key. For example, even if *n* − *t* participants lose their secret shadows, the remaining *t* participants are still able to recover the secret. Deploying TSS scheme can also improve the security of the system, as an attacker requires no less than *t* secret shadows to recover the secret. In traditional TSS schemes, after generating and distributing secret shadows, the dealer destroys the secret and exits. While in some TCSS schemes, the dealer is online until the secret is recovered by participants. For example, in Zhang et al.’s schemes, since the dealer needs to adjust the threshold and deal with the enrollment and disenrollment of the participant, he/she holds the secret and all secret shadows in the running phase until the secret is recovered. Thus, attackers only need to target the dealer in the attempt to obtain the secret. This results in single point of attack.

However, in our schemes, we use the combiner to take the dealer’s obligations in the running phase, and use two-variable one-way function to protect the information stored by the combiner. Thus, our schemes can update / revise the threshold in the running phase without the dealer’s involvement, which means our schemes is dealer-free. Meanwhile, our schemes protect the secret from being recovered when attackers have access to the information stored by the combiner. Hence, our schemes are more secure.

(3) Collusion attack

The (*t*, *n*) threshold secret sharing scheme can resist up to *t* − 1 collusion participants who have secret shadows. However, in the TCSS schemes of [21, 24, 28] based on the advance secret shadow technique, participants have both historical and current secret shadows after changing the threshold. Therefore, such schemes cannot resist attacks carried out by *t* − 1 colluding participants. Many schemes [21, 24, 28] require that all participants destroy the historical shadows if the threshold has been changed, but this may be unrealistic in practice (i.e. we are trusting the bad guys to do the right thing). Thus, Zhang et al. [16] proposed the first scheme (i.e. TCSS-B) designed to resist such collusion attack, by encrypting secret shadows and validating the new threshold with the corresponding key. In our schemes, two-variable one-way function is used to protect secret shadows from collusion attacks.

### Two-variable One-way Function

In this section, we introduce two-variable one-way function used in our schemes. Function *f*(*r*, *s*) is a two-variable one-way function, which maps variables *r* and *s* into a value with a fixed length. The features of *f*(*r*, *s*) are as follows [35]:

1. Given *r* and *s*, it is easy to compute *f*(*r*, *s*).
2. Given *s* and *f*(*r*, *s*), it is not feasible to compute *r*.
3. It is not feasible to compute *f*(*r*, *s*) for any *r* without *s*.
4. Given *s*, it is not feasible for *r*_*i*_ and *r*_*j*_ to satisfy *f*(*r*_*i*_, *s*) = *f*(*r*_*j*_, *s*), when *r*_*i*_ ≠ *r*_*j*_.
5. Given any pairs of (*r*_*i*_, *f*(*r*_*i*_, *s*)), it is not feasible to compute *s*.
6. Given any pairs of (*r*_*i*_, *f*(*r*_*i*_, *s*)), it is not feasible to compute *f*(*r*_*j*_, *s*), when *r*_*i*_ ≠ *r*_*j*_.

Assume that |*f*(*r*, *s*)| ≤ *q*, so *f*(*r*, *s*) ∈ *GF*(*q*). He and Dawson [36] proved the existence of two-variable one-way function, and also brought up the methods to construct it. For example, let *S* be a secure signature scheme. For a message *m*, the signature with secure key *k* is denoted by *S*(*k*, *m*). Let *H* be a universal one-way hash function whose existence is based on any one-to-one, one way function [37]. Two-variable one-way function *f*(*x*, *y*) can be constructed as *f*(*x*, *y*) = *H*(*S*(*x*, *y*)).

In our schemes, each participant *P*_*i*_ (1 ≤ *i* ≤ *n*) selects his/her own variable *s*_*i*_ (also referred to as real secret shadow), and the combiner has all variables *r*_1_, *r*_2_,⋯, *r*_*k*_ (*k* = *t*_max_ − *t*_min_ + 1 in DTCSS-A scheme and *k* = *N* in DTCSS-B scheme). By using two-variable one-way function, our schemes have the following advantages:

1. Collusion attack resistance: In our schemes, if and only if no less than current threshold (i.e., *t*_*j*_) participants wish to recover the secret, the combiner broadcasts the corresponding variant *r*_*j*_ to validate participants’ fake secret shadows. Colluding participants cannot obtain the historical shadows to recover the secret. Thus, our schemes can resist attacks carried out by *t*_*j*_ − 1 colluding participants who have both current and historical shadows.
2. Single point attack resistance: In our schemes, even if attackers obtain the information *r*_*j*_ stored in the combiner, they cannot compute the *f*(*r*_*j*_, *s*_*i*_) without *s*_*i*_. Thus, our schemes can avoid the limitation that attackers only need to target a single point in the attempt to obtain the secret.
3. Real secret shadow reusability: In our scheme, the real secret shadow can be reused in new secret reconstruction, thus, increasing the efficiency.

### Participants

There are *n* + 2 (*n* > 2) members in our schemes, including *n* participants, the dealer and the combiner. The obligations of these participants are as follows:

**Participants**: There are *n* participants who hold secret shadows. In the running phase, only equal to or greater than threshold value participants can cooperate to recover the secret.

**The dealer**: In the initialization phase, the dealer generates each participant’s advance secret shadows, and prepares for possible threshold change.

**The combiner**: In the running phase, the combiner adjusts the threshold value according to changes in the security policies and adversary structures prior to recovering the secret. Only if equal to or greater than threshold participants wish to recover the secret, then the combiner broadcasts the corresponding key to validate these participants’ current secret shadows. Once successfully validated, these participants can recover the secret.

In generally, there are only participants and dealers in (*t*, *n*) threshold secret sharing scheme. However, to avoid the dealer single point attack, we introduce a combiner. The combiner can be used to adjust threshold and validate participants’ corresponding secret shadows. We assume both dealer and combiner are trusted.

## Proposed Schemes

In this section, we introduce our schemes (i.e., DTCSS-A and DTCSS-B schemes). The notions and parameters used in our schemes are outlined in Table 1.

**Table 1:** Summary of Notations.

Notation	Meaning
*n*	Number of participants
*t*	Threshold value
*P*_*i*_	Participant *i*
*P*	Participant set, *P* = {*P*_1_, *P*_2_,⋯, *P*_*n*_}
*q*	A big prime number randomly chosen by the dealer, *q* > *n*
*S*	Domain of the secret, *S* = *GF*(*q*)
*s*	Secret, *s* ∈ *S*
*S*_*i*_	Domain of participant *P*_*i*_’s secret shadow, *S*_*i*_ = *GF*(*q*)
*s*_*i*_	Participant *P*_*i*_’s secret shadow, *s*_*i*_ ∈ *S*_*i*_
*T*	Domain of potential threshold
*t*′	New threshold in DTCSS-A scheme
*N*	Number of potential thresholds in DTCSS-B scheme
*h*(*x*)	A polynomial
*h*(*x*_*i*_)	Value of polynomial *h*(*x*) in a given *x*_*i*_
$y_{i}^{j}$	Participant *P*_*i*_’s *j*^*th*^ advance secret shadow
*ψ*_*i*_	Participant *P*_*i*_’s secret shadow updating function
*f*(*r*, *s*)	A two-variable one-way function
deg(⋅)	Operator is used for computing the degree of the polynomial
[*x*^*k*^]	Coefficient operator. If *h*(*x*) = ∑_*i*≥0_*a*_*i*_*x*^*i*^, then [*x*^*k*^] *h*(*x*) = *a*_*k*_.
[⋅]_*k*_	Polynomial operator. If *h*(*x*) = ∑_*i*≥0_*a*_*i*_*x*^*i*^, ${\left[h(x)\right]}_{k}=\sum_{i=0}^{k-1}a_{i}x^{i}$.

**Fig 1:**
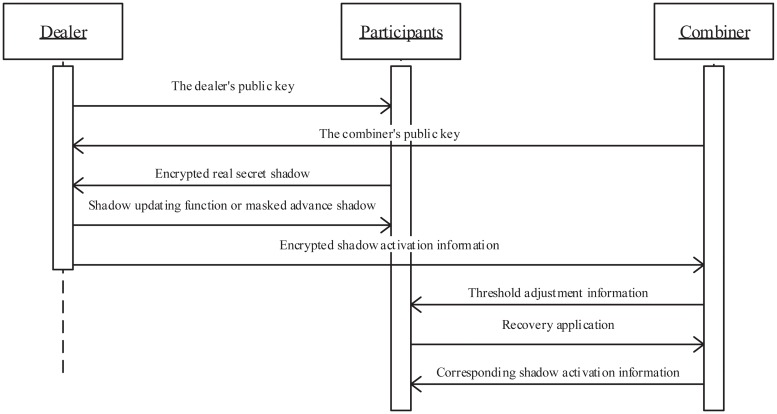
Sequence diagram of our schemes.

In our two DTCSS schemes, there are *n* + 2 (*n* > 2) members (i.e., *n* participants, the dealer and the combiner), and their message flows are shown in Fig 1. Specifically, real secret shadows (sent to the dealer by participants) and shadow activation information (sent to the combiner by the dealer) are sent using RSA-based technique, and other information is sent via broadcasting.

### (*t* → *t*′, *n*) Threshold Changeable Scheme

In this section, we present the dealer-free threshold changeable secret sharing scheme based on broadcasting (DTCSS-A), where the polynomial is used as the secret shadow updating function. This scheme is designed to convert a (*t*, *n*) scheme into a (*t*′, *n*) scheme, where *t*_min_ ≤ *t*′ ≤ *t*_max_.

Assume that the dealer knows the changeable threshold domain *T* = {*t*_min_, *t*_min_ + 1,⋯, *t*_max_}, where 2 ≤ *t*_min_ ≤ *t*_max_ ≤ *n* and $t_{\text{max}},t_{\min}\in Z$. Then, using the RSA-based technique, the dealer negotiates the real shadow with each participant. The dealer generates each participant’s advance secret shadows and secret shadow updating function, and publishes these functions. Prior to exiting, the dealer sends the information used to validate the secret shadow to the combiner. Based on any updates to the security policy and adversary structure, the combiner adjusts the threshold to a suitable value *t*′. If no less than *t*′ participants wish to recover the secret, then the combiner broadcasts $r_{t_{t'-t_{min}+1}}$. Therefore, these participants can recover the secret. DTCSS-A scheme consists of three phases as follows:

**1. Secret shadows negotiation phase**

In this phase, the dealer creates the notice table. Participants choose their own real secret shadows, and send them to the dealer using the underlying RSA technique. This phase has the following steps:

1. Notice table creation: To broadcast the message, the dealer creates a notice table, which can only be used for broadcasting information by the dealer and the combiner. Participants can obtain the information from the notice table, but they are unable to broadcast or modify the table.
2. Secret shadows negotiation initialization: Let *M*_1_ = *p*_1_ × *p*_2_ and *φ*(*M*_1_) = (*p*_1_ − 1) × (*p*_2_ − 1), where *p*_1_, *p*_2_ are big prime numbers chosen randomly by the dealer. The dealer chooses an integer *e*_1_ < *φ*(*M*_1_), which is co-prime with *φ*(*M*_1_). Then, the dealer computes the integer *d*_1_, such that $e_{1}d_{1}\equiv 1\left(\text{mod}\varphi \left(M_{1}\right)\right)$, and broadcasts {*e*_1_, *M*_1_} in the notice table. Similar, the combiner also generates his/her own {*e*_2_, *d*_2_, *M*_2_}, and broadcasts {*e*_2_, *M*_2_} in the notice table.
3. Real secret shadow generation and transfer: Each participant *P*_*i*_ (1 ≤ *i* ≤ *n*) chooses a real secret shadow *s*_*i*_ ∈ *S*_*i*_ randomly and sends *C*_*i*_ to the dealer, where $C_{i}={\left(s_{i}\right)}^{e_{1}}\text{ mod }M_{1}$. After receiving *C*_*i*_, the dealer recovers the real secret shadow $s_{i}={\left(C_{i}\right)}^{d_{1}}\text{ mod }M_{1}$, and then ensures all participants choose distinct secret shadows. If two or more participants choose the same secret shadow, they will be asked to choose their secret shadows again until all secret shadows are distinct.

**2. Initialization phase**

**Fig 2:**
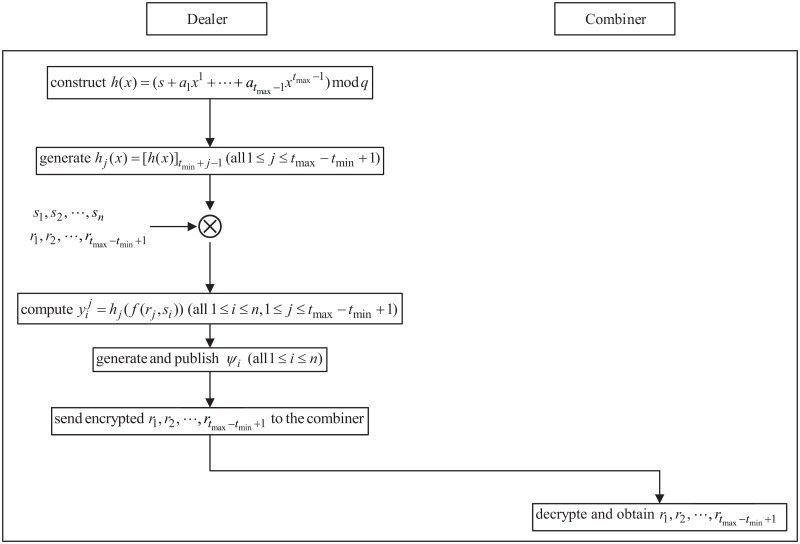
The work-flow of the initialization phase of DTCSS-A scheme.

In this phase, the dealer constructs the polynomial and generates each participant’s advance shadows. The work-flow of this phase is shown in Fig 2, and the working steps are as follows:

(1) Polynomial construction: To share a secret *s* ∈ *S*, the dealer constructs polynomial *h*(*x*) as

$$\begin{array}{c} h\left(x\right)=\left(s+a_{1}x^{1}+\cdots +a_{t_{\text{max}}-1}x^{t_{\text{max}}-1}\right)\;\mathrm{mod}\;q, \end{array}$$ (1)

where *a*_1_, *a*_2_,⋯, *a*_*t*_max_ − 1_ ∈ *GF*(*q*) are chosen randomly. Let *h*_*j*_(*x*) = [*h*(*x*)]_*t*_min_ + *j* − 1_ for all 1 ≤ *j* ≤ *t*_max_ −*t*_min_ + 1. Polynomial *h*_*j*_(*x*) can be generated by Algorithm 1.

Algorithm 1: Polynomial generator 1

Input: *h*(*x*), *j*, *t*_min_, *t*_max_

Output: *h*_*j*_(*x*)

 *h*_*j*_(*x*) = *h*(*x*);

 *d* = *t*_min_+*j* − 1;

 While *d* ≤ *t*_max_ − 1 do

  *c* = [*x*^*d*^]*h*(*x*);

  *h*_*j*_(*x*) = *h*_*j*_(*x*) − *cx*^*d*^;

  *d* = *d* + 1;

 end

(2) Secret shadow updating functions construction: The dealer selects *t*_max_ − *t*_min_ + 1 distinct and nonzero integers *r*_1_, *r*_2_,⋯, *r*_*t*_max_−*t*_min_+1_ ∈ *GF*(*q*) as keys. There is one-to-one correspondence between keys *r*_1_, *r*_2_, ⋯, *r*_*t*_max_−*t*_min_+1_ and thresholds *t*_min_, *t*_min_ + 1, ⋯, *t*_max_. Then, each participant’s advance secret shadows $y_{i}^{1},y_{i}^{2},\cdots ,y_{i}^{t_{\text{max}}-t_{\text{min}}+1}$ (1 ≤ *i* ≤ *n*) can be computed as

$$\begin{array}{c} y_{i}^{j}=h_{j}\left(x_{i}^{j}\right)\;\left(j=1,2,\cdots ,t_{\text{max}}-t_{\text{min}}+1\right), \end{array}$$ (2)

where $x_{i}^{j}=f\left(r_{j},s_{i}\right)$.

With *t*_max_ − *t*_min_ + 1 points $\left(x_{i}^{1},y_{i}^{1}\right),\left(x_{i}^{2},y_{i}^{2}\right),\cdots ,\left(x_{i}^{t_{\text{max}}-t_{\text{min}}+1},y_{i}^{t_{\text{max}}-t_{\text{min}}+1}\right)$, each participant’s secret shadow updating function *ψ*_*i*_ (1 ≤ *i* ≤ *n*) can be constructed as follows:

$$\begin{array}{c} \psi_{i}\left(x\right)=\sum_{j=1}^{t_{\text{max}}-t_{\text{min}}+1}y_{i}^{j}\prod_{k=1,k\neq j}^{t_{\text{max}}-t_{\text{min}}+1}\frac{x-x_{i}^{k}}{x_{i}^{j}-x_{i}^{k}}\;\mathrm{mod}\;q. \end{array}$$ (3)

Then, these functions are placed into notice table.

(3) Data transfer: To validate the new threshold in next phase, the dealer sends keys *r*_1_, *r*_2_, ⋯, *r*_*t*_max_−*t*_min_+1_ to the combiner before exiting. Note that these keys need to be encrypted by the combiner’s public key. Upon receiving this encrypted information, the combiner decrypts it.

**3. Running phase**

**Fig 3:**
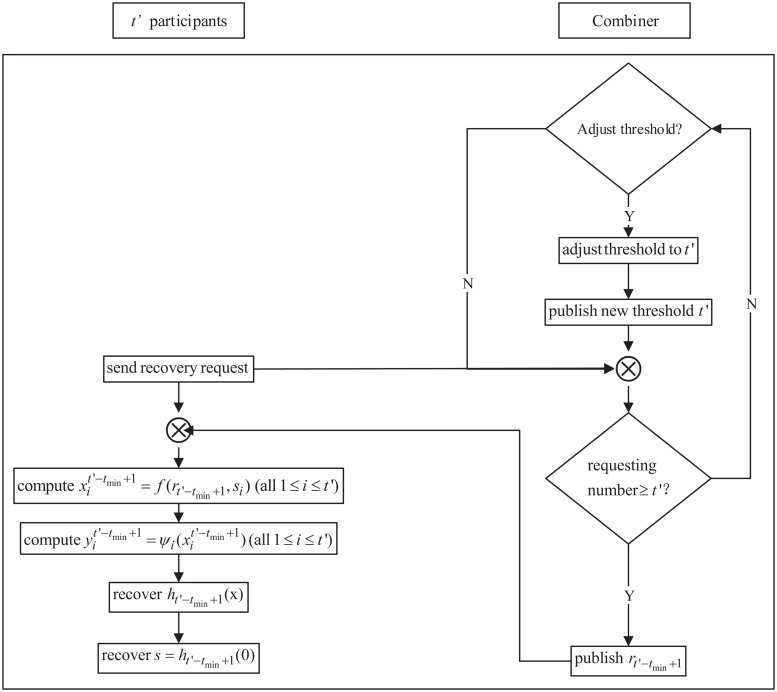
The work-flow of the running phase of DTCSS-A scheme.

In this phase, the security policy and adversary structure may change, which necessitates a threshold change prior to recovering the secret. Once the threshold has been revised, the secret can then be recovered with the most recently broadcasted threshold. The work-flow of the running phase is shown in Fig 3, and the working steps are as follows:

(1) Threshold adjustment: Based on the changes in the security policy and adversary structure, the combiner selects a suitable threshold *t*′ (*t*_min_ ≤ *t*′ ≤ *t*_max_), and inserts it into the notice table. The threshold can be adjusted many times before the secret is recovered.

(2) Shadow activation: If participants wish to recover the secret, they can send the recovery requests to the combiner. When *t*′ or more participants wish to recover secret *s*, the combiner broadcasts corresponding key $r_{t_{t'-t_{min}+1}}$. Then, each participant *P*_*i*_ (1 ≤ *i* ≤ *n*) can obtain her/his current secret shadow $y_{i}^{t^{\prime }-t_{\text{min}}+1}=\psi_{i}\left(x_{i}^{t^{\prime }-t_{\text{min}}+1}\right)$, where $x_{i}^{t^{\prime }-t_{\text{min}}+1}=f\left(r_{t^{\prime }-t_{\text{min}}+1},s_{i}\right)$.

(3) Secret recovery: Without loss of generality, we assume that *t*′ participants *P*_1_, *P*_2_, ⋯, *P*_*t*′_ wish to recover secret *s*. With *t*′ points $\left(x_{1}^{t^{\prime }-t_{\text{min}}+1},y_{1}^{t^{\prime }-t_{\text{min}}+1}\right),\left(x_{2}^{t^{\prime }-t_{\text{min}}+1},y_{2}^{t^{\prime }-t_{\text{min}}+1}\right),\cdots ,\left(x_{t^{\prime }}^{t^{\prime }-t_{\text{min}}+1},y_{t^{\prime }}^{t^{\prime }-t_{\text{min}}+1}\right)$, polynomial *h*_*t*′−*t*_min_+1_(*x*) can be recovered as

$$\begin{array}{c} h_{t^{\prime }-t_{\text{min}}+1}\left(x\right)=\sum_{i=1}^{t^{\prime }}y_{i}^{t^{\prime }-t_{\text{min}}+1}\prod_{j=1,j\neq i}^{t^{\prime }}\frac{x-x_{j}^{t^{\prime }-t_{\text{min}}+1}}{x_{i}^{t^{\prime }-t_{\text{min}}+1}-x_{j}^{t^{\prime }-t_{\text{min}}+1}}\;\mathrm{mod}\;q, \end{array}$$ (4)

where $x_{i}^{t^{\prime }-t_{\text{min}}+1}=f\left(r_{t^{\prime }-t_{\text{min}}+1},s_{i}\right)$ (1 ≤ *i* ≤ *t*′).

Then, we can recover secret *s* by computing *s* = *h*_*t*′−*t*_min_+1_(0).

### ({*t*_1_, *t*_2_,⋯, *t*_*N*_}, *n*) Threshold Changeable Scheme

In this section, we present our ({*t*_1_, *t*_2_,⋯, *t*_*N*_}, *n*) TCSS scheme (i.e., DTCSS-B scheme). Usually, *N* is a small integer. For example, when *N* = 3, {*t*_1_, *t*_2_, *t*_3_} correspond to the “low, middle, high” level of security in computers. Meanwhile, even if *t*_*N*_ is small, we always have *t*_*j*_ ≥ *j* for all 1 ≤ *j* ≤ *N*.

Let *t*_*k*_ (1 ≤ *k* ≤ *N*) be the value of initial threshold, and *t*_*j*_ (1 ≤ *j* ≤ *N*) be the value of the new threshold. Assume that the dealer knows *N* potential thresholds *t*_1_, *t*_2_,⋯, *t*_*N*_, where the threshold may be changed in the future and *t*_1_ < *t*_2_ < ⋯ < *t*_*N*_. Similar to the DTCSS-A scheme, the dealer negotiates the real shadow. The dealer generates each participant’s advance secret shadow. Prior to exiting, the dealer sends the information used to update and validates the secret shadow to the combiner. If the security policy or adversary structure changes, then the combiner adjusts the threshold to a suitable value *t*_*j*_, and broadcasts corresponding masked advance secret shadows. If no less than *t*_*j*_ participants wish to recover the secret, then the combiner broadcasts *r*_*j*_. Thus, these participants can recover the secret. The DTCSS-B scheme consists of three phases, namely: secret shadows negotiation, initialization and running.

**1. Secret shadows negotiation phase**

Similar to the DTCSS-A scheme, the dealer creates the notice table, and participants choose their own real secret shadows and send them to the dealer based on the underlying RSA technique.

**2. Initialization phase**

**Fig 4:**
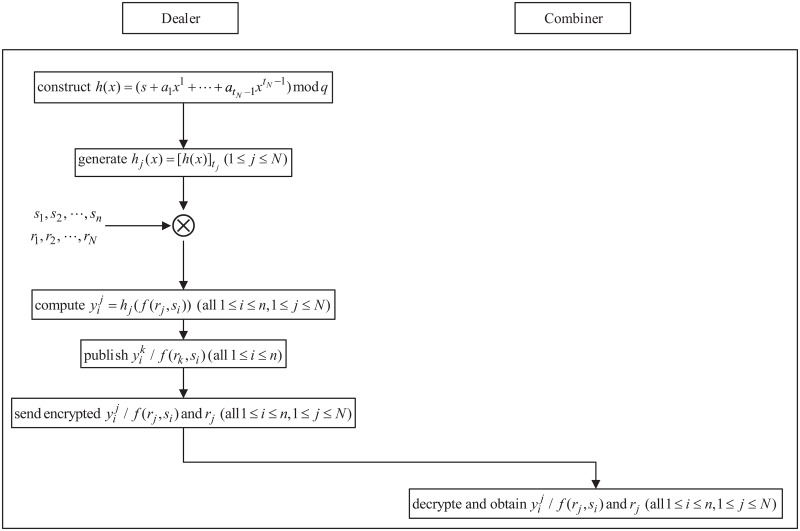
The work-flow of the initialization phase of DTCSS-B scheme.

In this phase, the dealer constructs the polynomial to protect the secret and generates the advance secret shadows for all participants. The work-flow of the running phase is shown in Fig 4, and the working steps are as follows:

(1) Polynomial construction: To share a secret *s*, polynomial *h*(*x*) is constructed as

$$\begin{array}{c} h\left(x\right)=\left(s+a_{1}x^{1}+\cdots +a_{t_{N}-1}x^{t_{N}-1}\right)\;\mathrm{mod}\;q, \end{array}$$ (5)

where *a*_1_, *a*_2_,⋯, *a*_*t*_*N*_−1_ ∈ *GF*(*q*) are chosen randomly. For all 1 ≤ *j* ≤ *N*, let polynomial *h*_*j*_(*x*) = [*h*(*x*)]_*t*_*j*__. Polynomial *h*_*j*_(*x*) can be generated by Algorithm 2.

Algorithm 2: Polynomial generator 2

Input: *h*(*x*), *j*, *t*_*j*_, *t*_*N*_

Output: *h*_*j*_(*x*)

 *h*_*j*_(*x*) = *h*(*x*);

 *d* = *t*_*N*_ − *t*_*j*_;

 While *d* > 0 do

  *c* = [*x*^*t*_*j*_+*d*−1^]*h*(*x*);

  *h*_*j*_(*x*) = *h*_*j*_(*x*) − *cx*^*t*_*j*_+*d*−1^;

  *d* = *d* − 1;

 end

(2) Advance secret shadows generation: The dealer chooses *N* distinct and nonzero integers *r*_1_, *r*_2_,⋯, *r*_*N*_ ∈ *GF*(*q*) as keys. There is one-to-one correspondence between keys *r*_1_, *r*_2_, ⋯, *r*_*N*_ and potential thresholds *t*_1_, *t*_2_, ⋯, *t*_*N*_. Each participant’s advance secret shadows $y_{i}^{1},y_{i}^{2},\cdots ,y_{i}^{N}$ (1 ≤ *i* ≤ *n*) are computed as follows:

$$\begin{array}{c} y_{i}^{j}=h_{j}\left(f\left(r_{j},s_{i}\right)\right)\;\left(j=1,2,\cdots ,N\right) \end{array}$$ (6)

Then, the dealer places masked advance secret shadows $y_{1}^{k}/f\left(r_{k},s_{1}\right),y_{2}^{k}/f\left(r_{k},s_{2}\right),\cdots ,y_{n}^{k}/f\left(r_{k},s_{n}\right)$ into the notice table.

(3) Data transfer: To validate the new threshold by the combiner in the next phase, the dealer sends $y_{1}^{j}/f\left(r_{j},s_{1}\right),y_{2}^{j}/f\left(r_{j},s_{2}\right),\cdots ,y_{n}^{j}/f\left(r_{j},s_{n}\right)$ (*j* = 1, 2,⋯, *N*) and *r*_1_, *r*_2_,⋯, *r*_*N*_ to the combiner, and then, he/she exits. Note that this information needs to be encrypted by the combiner’s the public key. After receiving this encrypted information, the combiner decrypts it.

**3. Running phase**

**Fig 5:**
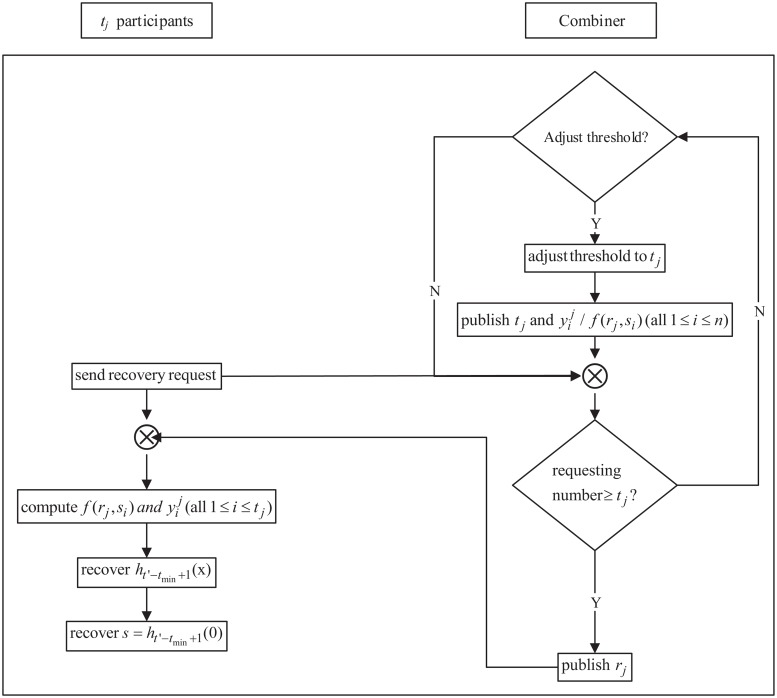
The work-flow of the initialization phase of DTCSS-B scheme.

In this phase, the security policy and adversary structure may change; thus, updating / revising the threshold is necessary before recovering the secret. The secret can then be recovered with the most recently broadcasted threshold. The work-flow of the running phase is shown in Fig 5, and the working steps are as follows:

(1) Threshold adjustment: Based on the changes in the security policy and adversary structure, the combiner selects a suitable threshold *t*_*j*_ (1 ≤ *j* ≤ *N*). Then, he/she places *t*_*j*_ and $y_{1}^{j}/f\left(r_{j},s_{1}\right),y_{2}^{j}/f\left(r_{j},s_{2}\right),\cdots ,y_{n}^{j}/f\left(r_{j},s_{n}\right)$ into the notice table. The threshold can be changed many times.

(2) Shadow activation: If participants wish to recover the secret, they can send the recovery requests to the combiner. When *t*_*j*_ or more participants wish to recover secret *s*, the combiner broadcasts key *r*_*j*_ to validate participants’ secret shadows, and then, each participant *P*_*i*_ (1 ≤ *i* ≤ *n*) can obtain the current secret shadow $\left(f\left(r_{j},s_{i}\right),y_{i}^{j}\right)$.

(3) Secret recovery: Without loss of generality, assume that *t*_*j*_ participants *P*_1_, *P*_2_, ⋯, *P*_*t*_*j*__ provide their current secret shadows $\left(f\left(r_{j},s_{1}\right),y_{1}^{j}\right),\left(f\left(r_{j},s_{2}\right),y_{2}^{j}\right),\cdots ,\left(f\left(r_{j},s_{t_{j}}\right),y_{t_{j}}^{j}\right)$. Polynomial *h*_*j*_(*x*) can be restructured as follows:

$$\begin{array}{c} h_{j}\left(x\right)=\sum_{i=1}^{t_{j}}y_{i}^{j}\prod_{k=1,k\neq i}^{t_{j}}\frac{x-f(r_{j},s_{k})}{f\left(r_{j},s_{i}\right)-f\left(r_{j},s_{k}\right)}\;\mathrm{mod}\;q \end{array}$$ (7)

Then, secret *s* can be recovered as *s* = *h*_*j*_(0).

## Analysis

### Security Analysis

In this section, we discuss and analyze the security of our schemes.

**Theorem 1**. In our schemes, each participant *P*_*i*_ (1 ≤ *i* ≤ *n*) is unable to obtain the valid secret shadow before the combiner broadcasts the key which corresponds to the current threshold.

**Proof**. Let the current threshold be *t*′ in the DTCSS-A scheme, and *t*_*j*_ in the DTCSS-B scheme. In the DTCSS-A scheme, according to the features of two-variable one-way function, each participant *P*_*i*_ (1 ≤ *i* ≤ *n*) is unable to obtain the valid secret shadow $\left(x_{i}^{t^{\prime }-t_{\text{min}}+1},y_{i}^{t^{\prime }-t_{\text{min}}+1}\right)$ before the combiner broadcasts *r*_*t*′−*t*_min_+1_, where $x_{i}^{t^{\prime }-t_{\text{min}}+1}=f\left(r_{t^{\prime }-t_{\text{min}}+1},s_{i}\right)$ and $y_{i}^{t^{\prime }-t_{\text{min}}+1}=\psi_{i}\left(x_{i}^{t^{\prime }-t_{\text{min}}+1}\right)$.

Similarly, in the DTCSS-B scheme, each participant *P*_*i*_ is unable to obtain the valid secret shadow $\left(x_{i}^{j},y_{i}^{j}\right)$ without *r*_*j*_, where $x_{i}^{j}=f\left(r_{j},s_{i}\right)$ and $y_{i}^{j}=\left(y_{i}^{j}/f\left(r_{j},s_{i}\right)\right)\times x_{i}^{j}$.

Thus, in our schemes, each participant *P*_*i*_ (1 ≤ *i* ≤ *n*) is unable to obtain the valid secret shadow prior to the combiner broadcasting the corresponding key.

The combiner broadcasts the key, if and only if the number of participants who wish to recover the secret is equal to or more than the current threshold value. Meanwhile, by Theorem 1, we know that each participant *P*_*i*_ (1 ≤ *i* ≤ *n*) is unable to compute the valid secret shadow without the key. Thus, participants do not have access to the historical secret shadow, or cannot undermine the security of our schemes using historical secret shadows.

**Theorem 2**. Less than current threshold value participants are unable to recover the secret.

**Proof**. Let the current threshold be *t*′ in the DTCSS-A scheme, and *t*_*j*_ in the DTCSS-B scheme. In the DTCSS-A scheme, without loss of generality, assume that *t*′−1 participants *P*_1_, *P*_2_,⋯, *P*_*t*′−1_ wish to recover the secret after the combiner broadcasts *r*_*t*′−*t*_min_+1_. Then, they can obtain *t*′−1 points $\left(x_{1}^{t^{\prime }-t_{\text{min}}+1},y_{1}^{t^{\prime }-t_{\text{min}}+1}\right),\left(x_{2}^{t^{\prime }-t_{\text{min}}+1},y_{2}^{t^{\prime }-t_{\text{min}}+1}\right),\cdots ,\left(x_{t^{\prime }}^{t^{\prime }-t_{\text{min}}+1},y_{t^{\prime }}^{t^{\prime }-t_{\text{min}}+1}\right)$, where $x_{i}^{t^{\prime }-t_{\min}+1}=f\left(r_{t^{\prime }-t_{\min}+1},s_{i}\right)$ and $y_{i}^{t^{\prime }-t_{\min}+1}=\psi_{i}\left(x_{i}^{t^{\prime }-t_{\min}+1}\right)$. Utilizing these points for each candidate point (*x*′, *y*′) (*x*′, *y*′ ∈ *GF*(*q*)), they can reconstruct one and only one polynomial $h_{t^{\prime }-t_{\min}+1}^{*}\left(x\right)$ of degree *t*′−1, which satisfy $h_{t^{\prime }{-}^{\prime }-t_{\min}+1}^{*}\left(x^{\prime }\right)=y^{\prime }$ and $h_{t^{\prime }-t_{\min}+1}^{*}\left(x_{i}^{j}\right)=y_{i}^{j}$ (1 ≤ *i* ≤ *t*′). Constructed in the same way, these possible polynomials are equally likely; thus, there is nothing an attacker can deduce about the real polynomial *h*_*t*′−*t*_min_+1_(*x*). Thus, they cannot recover secret *s* as in *s* = *h*_*t*′−*t*_min_+1_(0).

Similarly, in the DTCSS-B scheme, after the combiner has broadcasted *r*_*j*_ (1 ≤ *j* ≤ *N*), *t*_*j*_−1 participants are unable to recover the corresponding polynomial *h*_*j*_(*x*). Thus, they cannot recover secret *s* as in *s* = *h*_*j*_(0).

Thus, in our schemes, less than the current threshold value participants are unable to recover the secret.

By Theorem 1 and Theorem 2, we know that the secret can be recovered, if and only if equal to or greater than current threshold participants provide their valid secret shadows. Thus, our schemes are secure under changing threshold.

**Theorem 3**. Attackers are unable to recover secret *s* using only the information stored by the combiner.

**Proof**. In the DTCSS-A scheme, according to the features of two-variable one-way function, attackers who obtain keys *r*_1_, *r*_2_, ⋯, *r*_*t*_max_−*t*_min_+1_ cannot compute any participant’s secret shadow $\left(x_{i}^{j},y_{i}^{j}\right)$ (1 ≤ *i* ≤ *n*, 1 ≤ *j* ≤ *t*_max_ − *t*_min_ + 1) without *s*_*i*_, where $x_{i}^{j}=f\left(r_{j},s_{i}\right)$ and $y_{i}^{j}=\psi_{j}\left(x_{i}^{j}\right)$. Since *s*_*i*_ is only known by the dealer and participant *P*_*i*_, attackers cannot recover secret *s*.

Similarly, in the DTCSS-B scheme, even if attackers obtain *r*_1_, *r*_2_, ⋯, *r*_*N*_ and $y_{1}^{j}/f\left(r_{j},s_{1}\right),y_{2}^{j}/f\left(r_{j},s_{2}\right),\cdots ,y_{n}^{j}/f\left(r_{j},s_{n}\right)$ (*j* = 1, 2,⋯, *N*) stored by the combiner, they cannot obtain any participant’s secret shadows $\left(x_{i}^{j},y_{i}^{j}\right)$ (1 ≤ *i* ≤ *n*) without *s*_*i*_, where $x_{i}^{j}=f\left(r_{j},s_{i}\right)$ and $y_{i}^{j}=\left(y_{i}^{j}/f\left(r_{j},s_{i}\right)\right)\times x_{i}^{j}$.

Thus, attackers are unable to recover secret *s* using only the information stored by the combiner.

In no dealer-free schemes, the dealer may be compromised in the running phase, which results in the leakage of secrets and/or secret shadows. By Theorem 3, we know that our schemes can resist such attack. However, if the combiner’s information is stolen by attackers, the attackers only need to collude with no less than the minimum threshold participants to recover the secret. Note that the minimum thresholds are *t*_min_ and *t*_1_ in the DTCSS-A scheme and DTCSS-B scheme, respectively.

**Theorem 4**. In our schemes, attackers are unable to obtain any legitimate participant’s real secret shadow.

**Proof**. In the DTCSS-A scheme, assume that attackers wish to obtain the participant’ secret shadow *s*_*i*_ (1 ≤ *i* ≤ *n*), and they can obtain the exchanged information between the combiner and participant *P*_*i*_. Then, they can obtain *ψ*_*i*_, *r*_*t*′−*t*_min_+1_ and $\left(x_{i}^{t^{\prime }-t_{\min}+1},y_{i}^{t^{\prime }-t_{\min}+1}\right)$, where $x_{i}^{t^{\prime }-t_{\min}+1}=f\left(r_{t^{\prime }-t_{\min}+1},s_{i}\right)$ and $y_{i}^{t^{\prime }-t_{\min}+1}=\psi_{i}\left(x_{i}^{t^{\prime }-t_{\min}+1}\right)$. According to the features of two-variable one-way function, attackers are unable to compute *s*_*i*_ from *r*_*t*′−*t*_min_+1_ and $x_{i}^{t^{\prime }-t_{\min}+1}$.

Similarly, in the DTCSS-B scheme, attackers can obtain $r_{j},y_{i}^{j}/f\left(r_{j},s_{i}\right)$ and $\left(x_{i}^{j},y_{i}^{j}\right)$, where $x_{i}^{j}=f\left(r_{j},s_{i}\right)$ and $y_{i}^{j}=y_{i}^{j}/f\left(r_{j},s_{i}\right)\times x_{i}^{j}$. Thus, they cannot obtain *s*_*i*_ from *r*_*j*_ and $x_{i}^{j}$.

In summary, attackers cannot obtain any legitimate participant’s real secret shadow in our schemes.

By Theorem 4, we know that attackers cannot obtain any participant’s real secret shadow *s*_*i*_ (1 ≤ *i* ≤ *n*), so *s*_*i*_ can be reused in subsequent scheme.

### Comparative Summary

A comparative summary between our schemes and Zhang et al.’s schemes [16] are listed in Table 2.

**Table 2:** Comparative Summary.

	TCSS-A	DTCSS-A	TCSS-B	DTCSS-B
*NTL*	*t* < *t*′	*t*_min_ ≤ *t*′ ≤ *t*_max_	*t*′ ∈ {*t*_1_, *t*_2_,⋯, *t*_*N*_} (0 < *t*_*i* + 1_ − *t*_*i*_ < *t*_1_)	*t*′ ∈ {*t*_1_, *t*_2_,⋯, *t*_*N*_}
*NCP*	*t*′ − *t* + 1	1	*N*	1
*NRP*	$\frac{t^{\prime }-t+2}{2}$	1	$\frac{N+1}{2}$	1
*SSS*	(*t*′ − *t* + 1) log *q*	log *q*	*N* log *q*	log *q*
*BCS*	log *q*	log *q*	$\frac{N+1}{2}\log q$	(*n* + 1) log *q*

NTL: New threshold limitation; NCP: Number of constructing polynomials;

NRP: Number of recovering polynomials; SSS: Shadows storage space;

BCS: Broadcasting message space.

From Table 2, we observe that our schemes have following advantages:

**1. No limit on the threshold**

The new threshold *t*′ must satisfy *t* < *t*′ ≤ *n* in the TCSS-A scheme and *t*′ ∈ {*t*_1_, *t*_2_,⋯, *t*_*N*_} in the TCSS-B scheme, where 0 < *t*_*i*+1_ − *t*_*i*_ < *t*_1_ (*i* = 1, 2,⋯, *N* − 1). In both TCSS-A and TCSS-B schemes, the threshold can be changed only once. In our schemes, however, the new threshold *t*′ can be smaller than the initial threshold *t* in our DTCSS-A scheme, and *N* potential thresholds do not need to satisfy *t*_*i*+1_ − *t*_*i*_ < *t*_1_ (*i* = 1, 2,⋯, *N* − 1) in our DTCSS-B scheme. In addition, the threshold of our schemes can be changed more than once.

**2. Only one shadow storage requirement**

In the TCSS-A scheme, each participant *P*_*i*_ (1 ≤ *i* ≤ *n*) needs to store *t*′ − *t* + 1 secret shadows $s_{i}^{1},s_{i}^{2},\cdots ,s_{i}^{t^{\prime }-t+1}$ in threshold *t* and one secret shadow $s_{i}^{t^{\prime }-t+1}$ in threshold *t*′. In the TCSS-B scheme, all participants must store *N* secret shadows. However, in our schemes, each participant only has to store one secret shadow (i.e., *s*_*i*_), which results in significant savings for storage.

**3. Less computation**

In the TCSS-A scheme, in order to change the threshold, *t*′ − *t* + 1 polynomials *h*_1_(*x*), *h*_2_(*x*), ⋯, *h*_*t*′−*t*+1_(*x*) must be constructed in the initialization phase, and secret *s* is hidden in *t*′ − *t* coefficients of polynomial *h*_*t*′−*t*+1_(*x*), where deg(*h*_*t*′−*t*+1_(*x*)) = *t*′ − 1. If the threshold is changed, *t*′ or more participants can reconstruct polynomial *h*_*t*′−*t*+1_(*x*) directly. However, if the threshold is not changed, they have to determine polynomial *h*_1_(*x*) by polynomial interpolation, and then determine polynomials *h*_2_(*x*), *h*_3_(*x*), ⋯, *h*_*t*′−*t*+1_(*x*) in turn by computing:

$$\begin{array}{c} h_{j+1}\left(x\right)=h_{j}\left(x\right)+\frac{y_{i}^{j+1}-h_{j}\left(x_{i}\right)}{{\left(x_{i}\right)}^{t+j-1}}x^{t+j-1}\;\left(1\leq j\leq t^{\prime }-t\right), \end{array}$$ (8)

where $\left(x_{i},y_{i}^{j+1}\right)$ are provided by participant *P*_*i*_ who wants to recover the secret. Then, the secret can be obtained from the coefficients of polynomial *h*_*t*′−*t*+1_(*x*).

In the TCSS-B scheme, *N* polynomials *h*_1_(*x*), *h*_2_(*x*), ⋯, *h*_*N*_(*x*) must be constructed in the initialization phase. If the threshold is changed to *t*_*j*_ (1 ≤ *j* ≤ *N*), then they have to determine polynomial *h*_*j*_(*x*) using polynomial interpolation, and then, in turn, determine polynomials *h*_*j*+1_(*x*), *h*_*j*+2_(*x*), ⋯, *h*_*N*_(*x*) to recover the secret from polynomial *h*_*N*_(*x*).

However, in our schemes, only one polynomial needs to be constructed, and other corresponding polynomials can be obtained using polynomial operator [⋅]_*k*_. In addition, participants only need to recover polynomial *h*_*t*′−*t*_min_+1_(*x*) in our DTCSS-A scheme and polynomial *h*_*j*_(*x*) in our DTCSS-B scheme. Thus, the computational cost in our schemes is significantly lower than those of Zhang et al.’s schemes.

**4. Dealer-free**

Unlike our proposed schemes, Zhang et al.’s schemes require the dealer’s involvement in the running phase. By Theorem 3, we know that attackers are unable to recover the secret using only the information stored by the combiner. Thus, our schemes are more secure.

**5. Secret shadow reusability**

In Zhang et al.’s schemes, the secret shadow can be used to reconstruct only once, because those secret shadows are known to the participants who participate in recovering secret. However, in our schemes, the real secret shadow will not be leaked in recovering secret, which is demonstrated in Theorem 4. Thus, the real secret shadow can be reused to recover new secret, which results in increased efficiency.

### Application

In practice, the threshold may have to be adjusted if there are changes in the security policies and adversary structures prior to recovering the secret. Examples of changes that require threshold adjustment include: (1) an increase or decrease in the importance level of the secret; (2) a change in participant number (i.e., one or more participants joining or leaving the group); (3) a change in the level of mutual trust between participants; and (4) the leakage of some participants’ secret shadows. Our schemes can efficiently deal with these situations.

According to whether one or more secret shadows have been leaked, there are two kinds of situation in which the threshold needs to be adjusted:

1. No secret shadow leakage

This type includes the following 2 situation, i.e., an increase (decrease) in the importance level of the secret and a change in the level of mutual trust between participants. In these situations, all participants’ secret shadows are secure, so we only need to adjust threshold directly. For example, if the importance level of the secret increases (decreases), we only need to increase (decrease) the threshold.

2. one or more secret shadows leakage

This type includes the following two situations, namely: a change of participant number and the leakage of some participants’ secret shadows. In both situations, one or more participants’ secret shadows would be leaked (or could be easily stolen by attackers). Therefore, it is not sufficient to only adjust the threshold. Here, we use a change in the participant number as an example to discuss the threshold changeability for enrollment and disenrollment.

1. Enrollment: If some person joins the group in the running phase, then new secret shadows would be distributed to these enrolled participants. Therefore, the threshold does not need to be adjusted. Since our schemes are dealer-free, new secret shadows cannot be generated in the running phase. However, we can prepare redundant secret shadows for enrolled participants in the initialization phase. If any user wishes to be enrolled to the group, he/she will send the request to the combiner. Then, the combiner will distribute the redundant secret shadow to this requesting user. Note that the number of enrolled participants is less than or equal to the number of the redundant secret shadows.
2. Disenrollment: If some participants leave the group, then their secret shadows are useless to them. It is reasonable to assume that we should not rely on these departing participants to secure their secret shadows. Thus, we assume that these shadows could be easily compromised. In Zhang et al.’s scheme, if participants leave the group, then the dealer will broadcast their secret shadows and adjust the threshold. This could result in an increased risk since attackers only have to defeat the dealer to obtain all secret shadows. To avoid such a limitation, we propose two dealer-free schemes. If participants publish their shadows to all other participants (i.e. they leave the group), then the threshold will be adjusted by the combiner, without the involvement of a dealer. Therefore, the security of our schemes can be guaranteed. The detailed solutions dealing with the disenrollment are described below.

Let *k* be the number of disenrolled participants, *t* the current threshold value, and *P* = {*P*_1_, *P*_2_,⋯, *P*_*n*_} be the set of *n* participants. In the DTCSS-A scheme, if *k* participants broadcast their real secret shadows (e.g., when they leave the set *P*), then we obtain a new set *P*′ that does not include the disenrolled participants. The combiner would then adjust the threshold from *t* to *t* + *k*. In other words, the original (*t*, *n*) scheme is changed to a (*t* + *k*, *n* − *k*) scheme. Thus, *t* participants in set *P*′ can use their own real secret shadows and *k* published real secret shadows to recover the secret. Note that the actual minimum number of participants (i.e., *t*) for recovering the secret does not change. In this situation, the actual maximum changeable threshold is changed to *t*_max_ − *k*, and the maximum number of disenrolled participants is limited to *t*_max_ − *t*. In the DTCSS-B scheme, *k* is required to satisfy the condition (i.e., *t* + *k* ∈ {*t*_1_, *t*_2_,⋯, *t*_*N*_}), and no more than *t*_*N*_ − *t* participants can be allowed to leave the group.

We can also use the above described method to deal with secret shadow leakage. For example, if a participant’s secret shadow leaks, then this person can leave the group before rejoining. Through these operations, the security of the scheme can be guaranteed.

In summary, our schemes can efficiently deal with the situation in which the threshold needs to be adjusted. Thus, our schemes have broad and promising application potential.

## Conclusion

In this paper, we propose two improved dealer-free threshold changeable secret sharing schemes. By using two-variable one-way function, both schemes can resist collusion attacks launched by participants who hold both historical and current secret shadows. We also prove that our schemes can adjust the threshold safely, in the event that the security policy and adversary structure change. A comparative summary demonstrate that our schemes outperform Zhang et al.’s scheme, in terms of security and performance. Lastly, we discuss how our schemes can deal with situations where the threshold needs to be adjusted; thus, demonstrating the utility of our schemes in real-world deployments.

However, in order to minimize the size of broadcast message, the proposed DTCSS-A scheme requires significant computations to construct the secret shadow updating function. Meanwhile, in order to resist collusion attacks, our schemes can only validate participants’ secret shadow once. Thus, future research will include refining the scheme with the aim of improving its efficiency.
